# Comprehensive RNA-Seq transcriptomic profiling across 11 organs, 4 ages, and 2 sexes of Fischer 344 rats

**DOI:** 10.1038/sdata.2014.13

**Published:** 2014-06-24

**Authors:** Ying Yu, Chen Zhao, Zhenqiang Su, Charles Wang, James C Fuscoe, Weida Tong, Leming Shi

**Affiliations:** 1 Center for Pharmacogenomics, State Key Laboratory of Genetic Engineering and MOE Key Laboratory of Contemporary Anthropology, Schools of Life Sciences and Pharmacy, Fudan University, Shanghai 201203, China; 2 National Center for Toxicological Research, Food and Drug Administration, Jefferson, Arkansas 72079, USA; 3 Center for Genomics and Division of Microbiology & Molecular Genetics, School of Medicine, Loma Linda University, Loma Linda, California, 92350, USA; 4 Fudan-Zhangjiang Center for Clinical Genomics and Zhanjiang Center for Translational Medicine, Shanghai, 201203, China

## Abstract

The rat is used extensively by the pharmaceutical, regulatory, and academic communities for safety assessment of drugs and chemicals and for studying human diseases; however, its transcriptome has not been well studied. As part of the SEQC (i.e., MAQC-III) consortium efforts, a comprehensive RNA-Seq data set was constructed using 320 RNA samples isolated from 10 organs (adrenal gland, brain, heart, kidney, liver, lung, muscle, spleen, thymus, and testes or uterus) from both sexes of Fischer 344 rats across four ages (2-, 6-, 21-, and 104-week-old) with four biological replicates for each of the 80 sample groups (organ-sex-age). With the Ribo-Zero rRNA removal and Illumina RNA-Seq protocols, 41 million 50 bp single-end reads were generated per sample, yielding a total of 13.4 billion reads. This data set could be used to identify and validate new rat genes and transcripts, develop a more comprehensive rat transcriptome annotation system, identify novel gene regulatory networks related to tissue specific gene expression and development, and discover genes responsible for disease and drug toxicity and efficacy.

## Background and Summary

The rat is used extensively by the pharmaceutical, regulatory, and academic communities to test drug and chemical toxicities, to evaluate the mechanisms underlying drug effects, and to model human diseases (e.g., to evaluate drug efficacy). Next-generation sequencing technologies have revolutionized genomic research and allowed the genome and transcriptome of any organism to be explored without *a priori* assumptions and with unprecedented throughput^1–7,1–7,1–7,1–7,1–7,1–7,1–7^. Using RNA-Seq to catalogue the variations in the transcriptome between sexes and over the life span of the rat, from birth to old age, can provide insights into disease susceptibility, drug efficacy and safety, and toxicity mechanisms, and could ultimately improve the translation of pre-clinical findings to humans.

**Figure 1:**
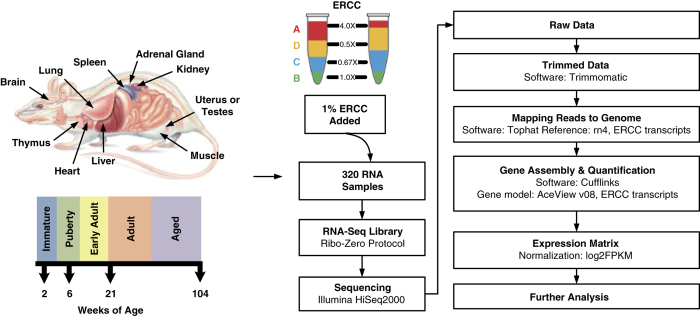
Schematic overview of rat transcriptomic BodyMap study design. Diagram of organs harvested from Fischer 344 rats and the selected four ages at which organs were harvested for RNA-Seq transcriptomic profiling. Total number of RNA samples: 10 organs per rat x 32 rats (2 sexes x 4 ages x 4 replicates) = 320 for RNA-Seq. Updated from Yu *et al.*^8^

Through the US Food and Drug Administration’s (FDA) SEQC (MAQC-III) consortium on next-generation sequencing quality control, 320 RNA-Seq libraries from 320 RNA samples derived from 16 females and 16 males from the Fischer 344 strain^8^ were constructed and sequenced. Ten organs (adrenal gland, brain, heart, kidney, liver, lung, muscle, spleen, thymus, and testis or uterus) were evaluated per rat at four ages, i.e., juvenile (2-weeks-old), adolescence (6-weeks-old), adult (21-weeks-old), and aged (104-weeks-old); and eight rats (four females and four males) were evaluated per age group (Figure 1). To assess inter-animal biological variations, four individual rats were tested for each of the 80 conditions (groups). To monitor the quality of the resulting RNA-Seq data, the External RNA Control Consortium (ERCC) spike-in controls, in an amount equivalent to about 1% of the mRNA in a test RNA sample^9^, were added before library construction. Approximately13.4 billion reads of 50 bp single-end RNA-Seq data were generated for this study, corresponding to an average of 41 million sequence reads per sample. Many transcripts that were previously only annotated in AceView^10^ based on cDNAs in GenBank and dbEST, were validated including 31,909 alternative spliced (AS) transcripts and 2,367 spliced non-coding genes/ncRNAs that were not annotated in RefSeq. This represents the first usage of large amounts of next-generation deep sequence data in rat to cross-validate AceView annotation. Next, a web-based, open-access rat transcriptome database (Data Citation 1) was constructed to catalogue the expression profiles for 40,064 AceView annotated genes and 65,167 transcripts measured in the 320 RNA samples. This unique and comprehensive RNA-Seq data set, accompanied by the online database searching capabilities, can serve as a useful resource for both academic biologists and pharmaceutical companies that utilize rats for assessing chemical safety profiles and for studying human diseases. An initial analysis of this data set has been published in a separate paper^8^.

## Methods

### Animals and organ collection

Female and male Fischer 344 rats (pair-housed under standard conditions) from the National Center for Toxicological Research (NCTR) of the US Food and Drug Administration animal breeding colony were euthanized by carbon dioxide asphyxiation at 2, 5, 6, 8, 15, 21, 52, 78, and 104 week-of-ages as previously described^8,11^. Organs (adrenal gland, brain, heart, kidney, liver, lung, muscle, spleen, thymus, and testes (males) or uterus (females)) from 2-week old (juvenile), 6-week old (adolescence), 21-week old (adult), and 104-week old (aged) rats were used in this study (Figure 1). At necropsy, whole organs were removed, quick-frozen in liquid N_2_, and stored at −80 °C for RNA extraction. Organs were harvested from four male and four female rats at each of the four ages. This study had ethical and scientific approval from the NCTR Institutional Animal Care and Use Committee. The rats were housed and euthanized according to NIH and institutional guidelines.

### RNA isolation

As described in the original data analysis paper^8^, each whole organ was individually ground (mortar and pestle, under continuous liquid N_2_ chilling) into a fine powder prior to RNA extraction, with the exception of liver, spleen, and gastrocnemius muscle for which approximately 100 mg was ground. Ground organ tissue was stored at −80 °C. Total RNA was extracted from approximately 30 mg of ground tissue by using the miRNeasy Mini Kit (Qiagen) according to the manufacturer’s protocol, including treatment with DNase. RNAs longer than 18 nucleotides were recovered with this method. RNA quality was evaluated with an Agilent 2100 Bioanalyzer (Agilent Technologies). All RNA samples had RNA integrity numbers (RINs) greater than 7.5, except for the eight spleen samples from rats of both sexes at 2 weeks-of-age (RIN: 2.2–5.1). Excluding these spleen samples, the average RIN was 9.2 for the other 312 RNA samples. RIN value and A260/A280 ratio of every sample were listed in Supplementary File 1.

### Construction of rRNA-depleted RNA-Seq libraries

To minimize batch effect during library construction and sequencing processes, the samples were processed in a randomized order (Supplementary File 1). An rRNA depletion protocol was coupled with the Illumina TruSeq RNA-Seq library protocol to construct the rat Bodymap RNA-Seq libraries. For each of the 320 RNA samples, one single RNA-Seq library was constructed. Total RNA (1 μg) spiked with 2 μl 1:100 diluted ERCC RNA spike-in control mix 1 or mix 2 (Life Technologies) was depleted of rRNA with the Ribo-Zero Nonmagnetic Kit (Epicentre). The rRNA-depleted RNA was purified using the RNA Clean & Concentrator Column (Zymo Research), which recovered all rRNA-depleted RNA, including small RNA. Then the TruSeq RNA Sample Preparation Kit (Illumina) was used, but skipped the Poly(A)^+^ selection step during library construction. The rRNA-depleted RNA was fragmented, followed by first and second strand cDNA synthesis. The cDNA was subject to end repair, adenylation of 3′ ends and adapter ligation. One of 12 unique indices in each randomized sample was used (for multiplexing). cDNA samples were purified using AMPure XP beads (Beckman Coulter) and then used in 15 cycles of PCR amplification (ABI GeneAmp PCR system 9700). The cDNA library quality and size distribution were checked using an Agilent Bioanalyzer and DNA 1000 chip. Library fragment sizes were between 200–500 bp, with a peak at approximately 260 bp. All libraries were quantified with a Qubit 2.0 Fluorometer (Life Technologies) (Supplementary File 1) and stored in non-sticky Eppendorf tubes (Life Technologies) at −20 °C.

### RNA-Seq library sequencing

RNA-Seq libraries were sequenced using Illumina’s TruSeq Cluster V3 flow cells and TruSeq SBS Kit V3 (Illumina). The 320 rat Bodymap libraries were clustered using TruSeq V3 flow cells, and sequenced (50 bp single-end read) on an Illumina HiSeq 2000 by Expression Analysis, Inc. Ten different RNA-Seq libraries (biological samples, randomized) were pooled together in equal amount and loaded in one single lane at a concentration of ~8.6 pM on two different flow cells for sequencing, giving two sequencing technical replicates for each biological sample. Eight flow cells were used for generating this data set. The number of reads for each of the 320 samples is shown in Supplementary File 1.

### Read mapping and quantification

The quality of the RNA-Seq data was firstly examined using the package FastQC (http://www.bioinformatics.babraham.ac.uk/projects/fastqc/). Duplication levels of the first 200,000 sequences in each sequencing sample were analyzed. Each sequence was tracked to the end of the file to give a representative count of the overall duplication level. To evaluate sequencing error, reads were mapped to the 92 ERCCs using Bowtie2 v2.1.0, with default parameter settings (- -end-to-end -D 15 -R 2 -N 0 -L 22 -i S,1,1.15)^12^. Mismatch was identified using in-house script (Supplementary File 2). Sequencing error rate was estimated by the rate of mismatch in all ERCC mapped reads. Based on sequence quality, GC content, duplication level and error rate of samples, parameters were set for sequence trimming using Trimmomatic^13^. Adapters and PCR sequences in the reads were clipped. The five bases at the beginning of sequencing reads were clipped. Bases off the end of a read were cut if below a threshold quality score of 20. Bases off the start of a read were cut if below a threshold quality score of 6. A sliding window of four bases was performed for cutting once the average quality within the window falls below a threshold quality score of 15. A sequence read was dropped if it was below 36 bases in length. The rat transcriptome annotation from AceView v08 was used (downloaded from ftp://ftp.ncbi.nih.gov/repository/acedb/ncbi_4_Sep08.rat.genes)^10^, which includes 40,064 unique genes, as the reference transcriptome. In addition, the rat genome UCSC rn4, downloaded from iGenome (ftp://igenome:G3nom3s4u@ussd-ftp.illumina.com/Rattus_norvegicus/UCSC/rn4/Rattus_norvegicus_UCSC_rn4.tar.gz), was used as the reference genome. Reads were aligned to the rat reference genome and AceView transcriptome with TopHat v2.0.4, allowing a maximum of two mismatches in the alignment^14^. The default parameter settings were used (- -bowtie2 -m 0 -i 50 -I 500000 -g 20 -x 60 -n 1 - -max-insertion-length 3 - -max-deletion-length 3). Alignment results were then processed using Cufflinks v2.0.2 for gene and transcript quantification (cuffdiff settings: -b -u - -no-diff -m 200 -s 80 -c 10 - -compatible-hits-norm) (Figure 1)^15^. Reads that did not align to the rat genome were converted to fastq format using bam2fastq (http://www.hudsonalpha.org/gsl/information/software/bam2fastq) and were then mapped to the 92 ERCCs that were then quantified using TopHat v2.0.4 and Cufflinks v2.0.2 with the same parameter settings described above. For samples with 2–3 technical replicates, average FPKM (Fragment Per Kilobase per Million mapped reads) values were used. To avoid infinite values, a value of one was added to the FPKM value of each gene before log_2_ transformation.

To estimate how complete and uniform a gene was covered by sequencing reads, the ‘gene body coverage percentage’ and the 3′/5′ coverage ratio, respectively, were calculated for the 20 ERCCs with the highest spike-in concentrations. Gene body coverage refers to the number of sequence reads mapped to a specific base within a gene and could be used to assess the degree of biases in sequencing coverage within a gene. Gene body coverage percentage was defined as formula (1):

(1)Genebodycoverage(%)=NumberofcoveredbasesNumberofbasesinagene×100%

A base in a gene was considered covered if at least one read was mapped to it. The 3′/5′ ratio of each ERCC transcript was calculated according to formula (2):

(2)3′/5′ratio=Averageperbasecoverageofthe50basesatthe3′endAverageperbasecoverageofthe50basesatthe5′end

The poly-A bases of a gene were excluded from counting. The average 3′/5′ ratio of the 20 ERCCs with the highest spike-in concentrations in one sample serves as the 3′/5′ ratio of the sample. The statistical analysis was based on R package^16^.

### Rat RNA-Seq transcriptomic BodyMap database

To facilitate community-wide use of this unique RNA-Seq data set, a web-based, open-access, user-friendly rat BodyMap transcriptomic profiling database (Data Citation 1) was created. The database entries were linked to many other widely-used databases, including AceView, GenBank, Entrez, Ensembl, RGD, UniProt, Gene Ontology, and Kyoto Encyclopedia of Genes and Genomes. Each gene with pre-defined expression features can be easily explored in the database. Users can query specific genes by using simple or complex search terms and can restrict the results to specific portions of the data set. For example, users can perform a query by (1) entering an Entrez ID or gene symbol in the search box; (2) selecting a region on the chromosome map, or entering a specific chromosome region in search box; (3) uploading the user’s own DNA sequences for BLAST homology search; or (4) just selecting items in the Browse page to view specific data. This transcriptomic data can be visualized intuitively in various plots based on many different comparisons as needed.

## Data Records

The rat RNA-Seq gene-expression data set (GSE53960) (Data Citation 2) consisting of 320 samples, as part of the SEQC project data sets (super series accession number: GSE47792) (Data Citation 3), has been deposited in GEO. In addition, the entire data set is downloadable from the web site specifically created for this study: http://pgx.fudan.edu.cn/ratbodymap/index.html (Data Citation 1). Raw reads were stored as SRA format (http://www.ncbi.nlm.nih.gov/books/NBK47537/). The fastq file names for each of the 320 samples can be found at Supplementary File 1.

## Technical Validation

### Quality metrics for RNA-Seq data

**Figure 2:**
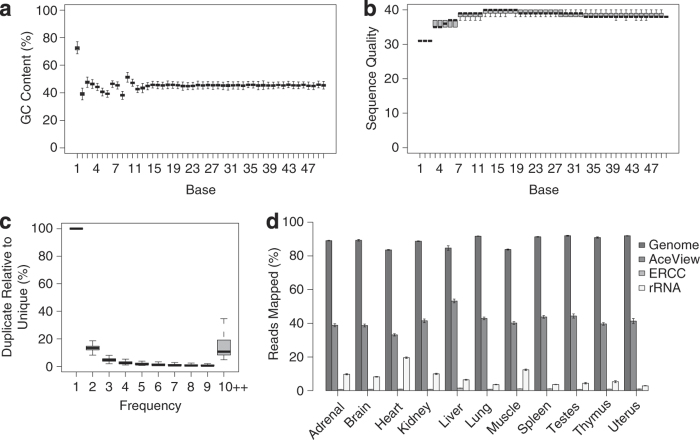
Quality assessment metrics for RNA-Seq data. Box plots representing (**a**) GC content (%) and (**b**) Phred quality score distribution over all reads across all samples in each base (i.e., sequencing cycle). The box and horizontal bar represent the interquartile range and median of the (**a**) GC content and (**b**) median of Phred quality score over all reads. (**c**) Box plot representing the percentage of reads (*y*-axis) that appear *N* times (*x*-axis) relative to the number of unique reads from each sequencing sample across all samples. (**d**) The percentage of reads mapped (ratio, Mean ±s.e., *n*=32 in normal organs or 16 in sexual organs) to genomic regions, AceView exons, ERCCs and rRNA in each organ.

For the purpose of estimating data quality, the GC content, sequence quality, duplication level, and mapping rate was examined for each sample. First, the GC content of the sequence data was examined that may impact the signal of interest (Figure 2a). Similar GC composition pattern with non-random bases in the first 12 bases near 5′-ends was observed in all samples, due to the use of random hexamers to prime the reverse transcription of RNA into double-stranded complementary DNA (dscDNA)^17^. Secondly, per base sequence quality across all samples was high, with a median quality score per base per sample >30 (Figure 2b). Thirdly, the complexity of the sampled reads was analyzed. On average, about 15% of reads were present more than ten times compared to the number of unique reads (Figure 2c), either from PCR duplications or the identical sequences from the same highly abundant gene. Based on these data quality measures, a set of parameters to trim sequence reads was selected (See Methods). After trimming, the reads to the rat AceView transcriptome, UCSC rn4 genome, and the ERCCs were mapped. On average, 88.5% of the reads were mapped to genomic regions, 41.7% to AceView exons, 8.2% to rRNA, and 0.92% to the ERCCs (Figure 2d). The mapping ratio for AceView exons, UCSC rn4 genome, rRNA, and ERCC are also listed in Supplementary File 1. Note that the ERCC mapping ratio for two samples was very low; it is likely that inadequate amount of ERCC mixes was added to these two RNA samples.

### Performance based on external RNA spike-in controls

**Figure 3:**
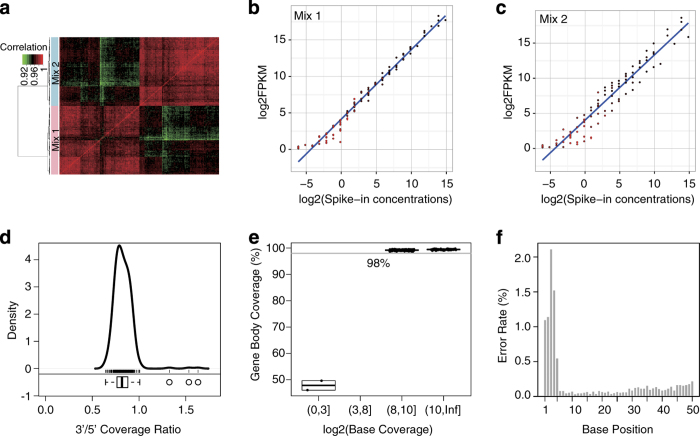
Quality assessment based on external RNA spike-in controls. (**a**) Heatmap and hierarchical clustering representing Pearson correlation between expression profiles of external RNA spike-in controls (ERCC) in 318 samples. Mix1 (pink) and Mix2 (light blue) are marked at the row side bar. The order of samples is identical in rows and columns. The plots of log2(FPKM) (Mean ±s.e., *n*=159) of ERCCs detected from samples spiked with ERCC Mix1 (**b**) and Mix2 (**c**) vs log2(spike-in concentrations). Linear fitting curve (blue) and error bar (red) are marked. (**d**) Kernel density and box plot representing 3′/5′-end coverage ratio across all samples. The average coverage of the 50 bases at the 3′- and 5′-ends of the 20 ERCCs with the highest spike-in concentrations is used to represent the coverage of the 3′- and 5′-ends in each sample. (**e**) Box plot for the gene body coverage ratio based on the 20 ERCCs with the highest spike-in concentrations. Four bins of the *x*-axis represent for average per-base coverage for the 20 ERCC sequences in each sample. *Y*-axis represents gene body coverage ratio, i.e., the percentage of bases that are sequenced at least one time. (**f**) Bar plot for single nucleotide sequencing error rate (*y*-axis) of each base along 5′- to 3′-end of a read (*x*-axis).

In order to assess the quality of RNA-Seq expression profiles across samples, either Mix1 or Mix2 of the 92 ERCC control sequences was spiked into each RNA sample. It was found that the expression profiles of the 92 ERCCs was highly correlated among samples (Figure 3a), indicating good reproducibility of the RNA-Seq data. Scatterplots of ERCC log2(FPKM) vs. log2(spike-in concentrations) showed an overall linear relationship between RNA-Seq detected signal and the true concentrations of the ERCC spike-in controls, in particular for controls with higher concentrations (Figures 3b, c). In addition, even though small disparities in all ERCCs were observed, ERCC at lower abundances showed more variability (Figures 3b, c). Furthermore, the sequence information of the ERCCs was used to estimate the gene body coverage and the sequencing error rate. Both the 3′/5′ coverage ratio (Supplementary File 3) and gene body coverage percentage was calculated for the 20 ERCCs with the highest spike-in concentrations (Figure 3d). The average 3′/5′ coverage ratio across all samples was 0.823, which means there was no 3′-bias. The slightly lower coverage at the 3′-end might have been a result of mapping bias of polyadenylation of ERCC sequences. Up to 98% of the bases of the 20 ERCCs were covered in all 318 samples, and the corresponding base coverage was larger than 2^8^, except for the two samples spiked with very low level of ERCC controls (Figure 3e). Finally, because there are no polymorphic sites in ERCC spike-in controls, it is possible to use the ERCC sequence information to estimate the sequencing error rate. It was found that the sequencing error rate was base-position dependent in all the samples. Specifically, the error rate was higher than 0.5% for the first five bases and remained as low as 0.1% for bases 6 to 28 before it started to increase gradually to ~0.2% at base 50 (Figure 3f). With a 5′-trimming preprocess, the overall sequencing error rate was ~0.1% in all samples (Supplementary File 3). The expected behavior of the external spike-in controls indicated that these data are of high quality.

### Reproducibility of biological replicates

The reproducibility of gene-expression profiles for the four biological replicates within the same sample group can be used as another metric for assessing the quality of the RNA-Seq data set. Thus, the pair-wise Pearson correlation coefficient (R) between any two of the four biological replicates within the same sample group was calculated based on the 40,064 genes, yielding six pair-wise *r* values per sample group. The mean *r* value and the standard error (s.e.) were calculated per group (*n*=6), yielding 80 mean *r* values and 80 s.e. values with a grand mean and s.e. of 0.9679 and 0.0014 (*n*=80), respectively, indicating a high-level of measurement consistency and reproducibility among biological replicates.

In Supplementary File 1 we provided detailed information about the 320 RNA samples used in the study (see Table 1 for a guide).

In Supplementary File 2 we provided the R script for identification of mismatch in ERCC sequences in this study.

In Supplementary File 3 we provided detailed information about the 664 fastq files generated in the study (see Table 2 for a guide).

## Usage Notes

An initial analysis of this data set has been published in a separate paper describing the general characteristics of the rat transcriptome across 11 organs, four ages, and two sexes^8^. This data set could be utilized, among many other possibilities, to effectively (1) identify and validate new rat genes and transcripts; (2) develop a more comprehensive annotation system for the rat transcriptome; (3) identify novel gene regulatory networks related to tissue specific gene expression and development; and (4) discover genes responsible for drug toxicity.

## Additional information

**How to cite this article:** Yu, Y. *et al.* Comprehensive RNA-Seq transcriptomic profiling across 11 organs, 4 ages, and 2 sexes of Fischer 344 rats. *Sci. Data* 1:140013 doi: 10.1038/sdata.2014.13 (2014).

## Supplementary Material



Supplementary File 1

Supplementary File 2

Supplementary File 3

## Figures and Tables

**Table 1:** Guide to Supplementary File 1

Column	Column Header	Explanation
1	Sample_ID	Sample identifier coding organ, sex, age, and replicate #
2	Libray_Processing_Order	The order in which an RNA sample or library was processed
3	RNA_Sample_ID	Sample identifier specifying the original serial sample ID
4	Flowcell	The flowcell number on which the sample was sequenced
5	Lane	The lane number on which the sample was sequenced
6	ERCC_Mix	Either ERCC Mix1 or Mix2 was added to the RNA sample
7	BarCode	The multiplexing barcode for the RNA sample
8	RNA_A260.A280_Ratio	A260/A280 ratio indicating RNA purity
9	RNA_RIN	RNA Integrity Number
10	Library_Con_ng.ul	Library concentration in ng μl^−1^
11	Organ	Full name of the organ from which the RNA was isoloated
12	Organ_Abbr_2chars	Two-character abbreviation for an organ
13	Organ_Abbr_3chars	Three-character abbreviation for an organ
14	Age_Week	Age (weeks) of the rat
15	Sex	Sex of the rat
16	Replicate	Replicate #
17	Sample_Name_Alt	Alternate name with two-character code for organ name
18	Genome_Ratio	The ratio of reads mapped to genome
19	Aceview_Ratio	The ratio of reads mapped to the AceView database
20	ERCC_Ratio	The ratio of reads mapped to the ERCCs
21	rRNA_Ratio	The ratio of reads mapped to rRNA
22	Total_Reads	The number of total reads collected on an RNA sample

**Table 2:** Guide to Supplementary File 3

Column	Column Header	Explanation
1	Filenames	A unique filename coding organ, sex, age, replicate #, flowcell, lane, and library pool
2	baseCov	Per-base coverage for the top 20 ERCC sequences
3	5′ normCov	Length normalized per-base coverage of 50 bases in 5′ end across top 20 ERCC sequences
4	3′ normCov	Length normalized per-base coverage of 50 bases in 3′ end across top 20 ERCC sequences
5	5′/3′ Cov Ratio	3′ bias based on normCov
6	Gap%	Percentage of bases that were not sequenced in the top 2 ERCC sequences
7	Base Mismatch Rate	Sequencing error rate in preprocessed sequencing data
